# Factors associated with scientific misconduct and questionable research practices in health professions education

**DOI:** 10.1007/s40037-019-0501-x

**Published:** 2019-03-26

**Authors:** Lauren Maggio, Ting Dong, Erik Driessen, Anthony Artino Jr.

**Affiliations:** 1 Division of Health Professions Education and Department of Medicine, F. Edward Hébert School of Medicine, Uniformed Services University of the Health Sciences in Bethesda, Bethesda, MD USA; 2 Department of Medicine, F. Edward Hébert School of Medicine, Uniformed Services University of the Health Sciences in Bethesda, Bethesda, MD USA; 3 Department of Educational Development and Research, Faculty of Health, Medicine and Life Sciences of Maastricht University, Maastricht, The Netherlands

**Keywords:** Questionable research practices, Research ethics, Misconduct, Survey

## Abstract

**Introduction:**

Engaging in scientific misconduct and questionable research practices (QRPs) is a noted problem across fields, including health professions education (HPE). To mitigate these practices, other disciplines have enacted strategies based on researcher characteristics and practice factors. Thus, to inform HPE, this study seeks to determine which researcher characteristics and practice factors, if any, might explain the frequency of irresponsible research practices.

**Method:**

In 2017, a cross-sectional survey of HPE researchers was conducted. The survey included 66 items adapted from three published surveys: two published QRP surveys and a publication pressure scale. The outcome variable was a self-reported misconduct score, which is a weighted mean score for each respondent on all misconduct and QRP items. Statistical analysis included descriptive statistics, reliability and correlation analysis, and multiple linear regression modelling.

**Results and Discussion:**

In total, 590 researchers completed the survey. Results from the final regression model indicated that researcher age had a negative association with the misconduct score (*b* = -0.01, *β* = -0.22, *t* = -2.91, *p* <0.05), suggesting that older researchers tended to report less misconduct. On the other hand, those with more publications had higher misconduct scores (*b* = 0.001, *β* = 0.17, *t* = 3.27, *p* < 0.05) and, compared with researchers in the region of North America, researchers in Asia tended to have higher misconduct scores (*b* = 0.21, *β* = 0.12, *t* = 2.84, *p* < 0.01). In addition, compared with those who defined their work role as clinician, those who defined their role as researcher tended to have higher misconduct scores (*b* = 0.12, *β* = 0.13, *t* = 2.15, *p* < 0.05). Finally, publication pressure emerged as the strongest individual predictor of misconduct (*b* = 0.20, *β* = 0.34, *t* = 7.82, *p* < 0.01); the greater the publication pressure, the greater the reported misconduct. Overall, the explanatory variables accounted for 21% of the variance in the misconduct score, with publication pressure accounting for 10% of the variance in the outcome, above and beyond the other explanatory variables. Although correlational, these findings suggest several researcher characteristics and practice factors that could be targeted to address scientific misconduct and QRPs in HPE.

**Electronic supplementary material:**

The online version of this article (10.1007/s40037-019-0501-x) contains supplementary material, which is available to authorized users.

## What this paper adds

Researchers engaging in scientific misconduct and questionable research practices (QRPs) is a noted problem across many fields, including health professions education (HPE). To mitigate these practices, fields outside HPE have implemented strategies based on researcher characteristics and practice factors. This study investigated associations between HPE researcher characteristics (e. g., gender, years of practice); publication pressure; and frequency of self-reported scientific misconduct and QRPs. Findings suggest that publication pressure, as well as several researcher characteristics, explain significant variance in investigator misconduct and QRPs in HPE. These findings provide an evidence base from which HPE might tailor strategies to address irresponsible research practices.

## Introduction

In health professions education (HPE), a recent international survey of researchers reported that 90% of almost 600 respondents admitted to engaging in scientific misconduct and questionable research practices (QRPs). These practices ranged from serious infractions, such as data fabrication and plagiarism, to less severe violations, like excessive self-citations, inappropriate data storage, and so-called ‘salami slicing’ [1]. Similarly, a study of authorship in HPE unearthed a number of questionable practices, such as granting honorary authorship and denying authorship to individuals who deserved it [2]. These recent studies highlight a potential problem in HPE, a problem that can harm the research enterprise by wasting limited resources, damaging the scientific record, disadvantaging unwitting researchers, and setting a poor example for trainees [3].

To inform approaches that might mitigate research misconduct and QRPs, researchers in biomedicine [4, 5], psychology [6], and business [7] have attempted to identify relationships between personal characteristics, practice factors, and researchers’ engagement in irresponsible research behaviours. To date, these studies have considered variables such as gender, career stage, geographical location, primary research methodology used, and pressure to publish [6, 8]. For example, based on a self-report survey of biomedical researchers, Tijdink and colleagues found that publication pressure and early career stage were associated with an increased likelihood of engaging in QRPs [5]. In another study that examined authors of retracted biomedical journal articles, investigators found that junior researchers were more likely to have had a paper retracted; however, this study did not identify publication pressure as a predictor of paper retraction [8]. These findings and others have led for calls for increased transparency in research and the utilization of open science practices in order to mitigate misconduct and QRPs [9].

Although we now have a sense of the frequency of scientific misconduct and QRPs in HPE [1], we do not yet know which personal characteristics or practice factors may explain a researcher’s engagement in these irresponsible behaviours. Additionally, as a relatively new field ‘without a roadmap to navigate these challenges’ [10], HPE is populated by multidisciplinary researchers who draw upon and combine varied backgrounds (e. g., biomedicine, psychology, education). Therefore, we propose that related findings from other fields may not generalize to the HPE context. This dearth of knowledge hampers our efforts to address misconduct and QRPs in HPE. Thus, the present study aims to address this gap by examining the associations between HPE researcher characteristics (e. g., gender, years of practice, academic background, geographic location, and primary methodology used in research); a potentially important practice factor (publication pressure); and the frequency of self-reported scientific misconduct and QRPs.

## Method

In 2017, we conducted an anonymous, cross-sectional survey of HPE researchers as a component of a larger program of research on responsible research practices. The survey’s purpose was twofold: (1) to measure the frequency of self-reported misconduct and QRPs in HPE and (2) to determine which researcher characteristics and practice factors, if any, might explain the frequency of these irresponsible behaviours. In 2018, we chose to address each of these purposes separately, as we felt that each warranted their own exploration and analysis, and each told a different yet equally valued story [11]. Therefore, we first published an article detailing the creation and execution of the survey, and we reported on the frequency of research misconduct and QRPs, thus confirming that these practices are present in HPE [1]. In the current article, we now focus our attention to the relationships between researcher characteristics, practice factors, and irresponsible research practices, including both deliberate scientific misconduct and QRPs, to help unearth important characteristics and practices that might be targeted for intervention. Ethical approval for this study was granted by the Ethical Review Board Committee of the Netherlands Association for Medical Education (Dossier #937).

### Survey development

We developed a 66-item survey by adapting three published survey instruments [4, 12, 13] (see online Supplementary Electronic Material, Appendix 1). The scientific misconduct and QRP items were slightly modified from the original instruments to improve clarity and relevance to the HPE context. Further details about this adaptation process, including the 19 expert reviews that were conducted, have been published previously [1].

The survey’s first set of items asked participants to indicate how often, if ever, they had engaged in research misconduct or other QRPs. The response options were *never, once, occasionally, sometimes, frequently*, or *almost always*. Participants also had the option of selecting *not applicable to my work*. In addition to questions on irresponsible research behaviours, participants responded to a nine-item, publication pressure scale, adapted from Tijdink [13]. The publication pressure scale utilized a five-point, bipolar, agreement-response scale, as originally designed by the authors [13]. The final survey also included 13 demographic items.

### Sampling and survey distribution procedures

We created our sample using two separate approaches: the first approach was based on published journal articles and the second on social media. First, we created a ‘curated sample’ of HPE researchers who had published in 20 HPE journals between 2015-2016 (See Supplementary Electronic Material, Box 1 for listing of all journals). To identify authors, we searched these 20 journals via Web of Science, Scielo (a Latin American database), African Journals Online, and Asia Journals Online. From these articles, we extracted all author email addresses and removed any duplicate emails resulting in a sample of 1,840 unique HPE researchers. Survey invitations were distributed via Qualtrics, an online survey tool (Qualtrics, Provo, Utah), in four waves on 13 November 2017, 20 November 2017, 27 November 2017, and 11 December 2017. Of the 1,840 email invitations sent, 199 bounced back as undeliverable, reducing the number of potential respondents to 1,641.

Next, on 11 December 2017, we created the ‘social media sample’ by posting a link to the survey to our personal accounts on Twitter and Facebook. All survey responses obtained from the social media links were tracked separately from the curated sample. Respondents in the social media sample were given the option to select ‘I have already completed this survey’ on the survey welcome page to help us avoid duplicate responses.

### Statistical analyses

For the curated sample, we calculated the response rate based on the definitions provided by the American Association for Public Opinion Research [14]. To assess potential nonresponse bias in the curated sample, we used wave analysis to calculate a nonresponse bias statistic (for additional details on this study’s application of wave analysis see Artino [1]).

### Measures

The outcome measure was a misconduct score, which is a weighted mean score for each respondent on all 43 misconduct and QRP items [5]. Because the misconduct and QRP items varied greatly on the dimension of ‘severity’ (i. e., the degree to which the behaviour might distort or damage science), we created a weighted frequency score using weightings from Bouter et al.’s (2016) ‘impact on validity’ rankings [12]. For example, the item ‘fabricated data’ had the highest severity weighting of 4.63, whereas the item ‘added one or more authors to a paper who did not qualify for authorship (so-called honorary authorship)’ had the lowest severity weighting of 2.07.

The explanatory measures were the other survey variables collected, including variables measured on a nominal scale (gender, geographical region of work, academic rank, type of research, and work role [clinician, researcher, or other]), and variables measured on a ratio scale (age, publication pressure composite score, number of publications, percentage of work time doing health professions or medical education, and years involved in health professions or medical education). The publication pressure composite score was calculated as an unweighted mean score for the nine items that comprised the publication pressure scale. Selection of these explanatory variables was based on the ethical research literature from other fields [5, 6, 8].

The statistical analysis consisted of descriptive statistics of the measured variables, internal consistency reliability analysis of the nine-item publication pressure scale, correlation analysis, and hierarchical multiple linear regression, using the misconduct score as the outcome variable. For the descriptive statistics, we report mean, standard deviation, and range if a measure is on a ratio scale and frequency count and valid percentage if a measure is on a nominal scale. We also conducted bivariate Pearson correlation analysis for all variables except those that were dummy coded (i. e., region of work, academic rank, type of research, and work role). For multiple linear regression modelling, we checked the explanatory variables for excessive multicollinearity and performed residual analysis followed by variable transformation to improve model fit. Finally, we fit a three-step hierarchical multiple linear regression model, entering the explanatory variables in three blocks. The first block consisted of age, gender, and years involved in health professions or medical education. The second block consisted of number of publications, region of work, academic rank, type of research, work role, and percentage of work time doing health professions or medical education. And the third block included the publication pressure composite score. The purpose of the regression analysis was to estimate, with some accuracy, the relationships between the explanatory and outcome variables. The statistical analyses were conducted in IBM SPSS 24.0 (IBM Corporation, New York, NY).

## Results

Four hundred and sixty-three respondents from the curated sample completed at least a portion of the survey, resulting in a response rate of 28.2% (potential respondents *n* = 1,641). Based on the wave analysis, we identified a nonresponse bias statistic of 0.36. Since we utilized a six-point, frequency-response scale, this bias statistic represents a 6% difference between the scores from respondents in the last wave compared with those in the first. For practical purposes, this small difference is unlikely to have a meaningful effect on the results [15].

Our social media recruitment netted an additional 127 responses. Using a multivariate analysis of variance, we compared the curated sample with those in the social media sample and found statistically significant differences *F*(5, 524) = 6.67, *p* < 0.01. Post-hoc analyses indicated that those in the curated sample were slightly older (*M* = 47.4 years) and more experienced as HPE researchers (*M* = 11.0 years) than those in the social media sample (*M* = 40.7 years; *M* = 7.5 years). However, the groups were similar in the mean number of articles published and percentage of work dedicated to HPE research. Thus, because we aimed to understand the relations between HPE researcher characteristics, practice factors, and scientific misconduct and QRPs among a diverse, international sample, we combined the responses from the two groups and analyzed all participant responses together.

Descriptive statistics for the variables measured on a ratio scale are displayed in Tab. 1 and those on a nominal scale reported in Tab. 2. Results from the reliability analysis of the nine publication pressure items indicated that the item scores had a reasonably high internal consistency reliability coefficient (Cronbach’s alpha = 0.83)[16].

**Table 1:** Descriptive statistics of ratio scale variables

Variable	Mean	SD	Range
Weighted misconduct frequency score	0.84	0.83	0–4.76
Age	46.03	11.64	23–87
Publication pressure composite score	2.97	0.79	1–5
Number of publications	40.08	54.98	0–600
Percentage of work time doing health professions or medical education	27.32	23.69	0–100
Years involved in health professions or medical education	14.91	9.67	0–54

**Table 2:** Descriptive statistics of the nominal scale variables

Variable	Category	Frequency	Valid percentage
Gender	Female	305	55.4
	Male	246	44.6
Region of work	North America	246	45.1
	Europe	137	25.1
	Australia/New Zeeland	42	7.7
	Asia	37	6.8
	Africa	53	9.7
	South America	18	3.3
	Others	12	2.2
Academic rank	Trainee	89	16.3
	Junior faculty	163	29.9
	Senior faculty	255	46.8
	Other	38	7.0
Type of research	Quantitative	149	27.2
	Qualitative	119	21.7
	Mixed methods	280	51.1
Work role	Clinician	136	24.7
	Researcher	174	31.6
	Administrator/program director, teacher, or others	240	43.6

Results of two-way Pearson correlation analysis for the variables on a ratio scale are displayed in Tab. 3. As indicated in the table, publication pressure (*r* = 0.35, *p* < 0.01) and percentage of work time doing HPE research (*r* = 0.10, *p* < 0.05) were both positively correlated with the misconduct score. On the other hand, age (*r* = −0.19, *p* < 0.01) and years involved in HPE (*r* = -0.12, *p* < 0.01) were both negatively correlated with the misconduct score.

**Table 3:** Pearson correlation coefficients of all variables except dummy coded nominal variables (region of work, academic rank, type of research, and work role) (*N* = 590)

	Age	Gender	Years involved in health professions or medical education	Number of publications	Percentage of work time doing health professions or medical education	Publication pressure composite score†	Misconduct score
Age	–	−0.09*	0.81**	0.48**	−0.09*	−0.34**	−0.19**
Gender (male = 1; female = 2)		–	−0.10*	−0.20**	0.09*	0.01	−0.10*
Years involved in health professions or medical education			–	0.50**	−0.05	−0.30**	−0.12*
Number of publications				–	0.10*	−0.24**	0.05
Percentage of work time doing health professions or medical education					–	0.14**	0.10*
Publication pressure composite score						–	0.35**

^†^The publication pressure composite score was calculated as an unweighted, mean score for the nine items that comprised the publication pressure scale (Cronbach’s alpha = 0.83)

*P < 0.05; **P < 0.01

The results of the three-step hierarchical multiple linear regression are presented in Tab. 4. When the weighted misconduct score was used as the outcome variable, the normal probability plot of standardized residual indicated unsatisfactory model fit. Thus, we transformed the variable by taking its square root. The normal probability plot of standardized residual after transformation indicated a much improved model fit. Moreover, the variance inflation factor for all of the explanatory variables ranged from 1.06 to 3.52, suggesting very little multicollinearity [17].

**Table 4:** Three-step hierarchical multiple linear regression modeling. The outcome variable is the square root of weighted misconduct score (*N* = 590)

Explanatory variable	Block 1			Block 2			Block 3		
	Unstandardized regression coefficient	Standardized regression coefficient	*t*-test statistic	Unstandardized regression coefficient	Standardized regression coefficient	*t*-test statistic	Unstandardized regression coefficient	Standardized regression coefficient	*t*-test statistic
***Block 1***									
Age	**−0.01**	**−0.28**	−3.86**	**−0.01**	**−0.31**	−3.95**	**−0.01**	**−0.22**	−2.91**
Gender (Male = 1; Female = 2)	−0.07	−0.07	−1.72	−0.06	−0.06	−1.33	−0.04	−0.05	−1.12
Years involved in health professions or medical education	0.003	0.07	1.03	0.002	0.05	0.68	0.003	0.06	0.84
***Block 2***									
Number of publications				** 0.001**	** 0.13**	2.37*	** 0.001**	** 0.17**	3.27**
Region of work (North America as reference group^ξ^)									
Europe				0.05	0.05	1.05	0.03	0.02	0.54
Australia/NZ				−0.05	−0.03	−0.68	−0.12	−0.07	−1.60
Asia				** 0.28**	** 0.16**	3.53**	** 0.21**	** 0.12**	2.84**
Africa				0.08	0.05	1.19	0.04	0.03	0.57
South America				0.11	0.04	0.93	0.10	0.04	0.93
Academic Rank (junior faculty as reference group)									
Trainee				0.005	0.004	0.08	0.04	0.03	0.65
Senior faculty				−0.005	−0.01	−0.10	−0.004	−0.01	−0.09
Type of research (quantitative methods as reference group*)									
Qualitative				0.04	0.03	0.63	0.06	0.06	1.15
Mixed-methods				−0.03	−0.03	−0.67	−0.02	−0.03	−0.56
Work role (clinician as reference group)									
Researcher				0.11	0.11	1.84	** 0.12**	** 0.13**	2.15*
Other				0.02	0.02	0.36	0.03	0.03	0.60
Percentage of work time doing health professions or medical education				0.0004	0.02	0.40	−0.001	−0.03	−0.72
***Block 3***									
Publication pressure composite score							**0.20**	** 0.34**	7.82**
***Model summary statistics***									
*R*^*2*^* c*hange for Block	0.052**			0.058**			0.097**		
Total *R*^*2*^	0.052			0.109			0.206		

*P < 0.05; **P <0.01

ξThese groups were selected as the reference groups because they represented the largest respondent groups in their respective categories.

All the regression coefficient estimates in Tab. 4 were calculated using the square root of weighted misconduct score as the outcome variable. In block 1, researcher age had a significant negative association with the outcome variable (*b* = -0.01, *β* = -0.28, *t* *=* -3.86, *p* <0.01); this suggests that older researchers tended to have lower misconduct scores. In total, the three variables entered in block 1 explained 5% of the variance in the outcome. In block 2, number of publications (*b* = 0.001, *β* = 0.13, *t* = 2.37, *p* < 0.05) had a significant positive association with the outcome when both age and years involved in health professions or medical education were controlled for in the preceding block. Compared with researchers in the region of North America, researchers in Asia tended to have higher misconduct scores (*b* = 0.28, *β* = 0.16, *t* = 3.53, *p* < 0.01). In addition, compared with those who defined their work role as clinician, those who defined their work role as researcher tended to have higher misconduct scores (*b* = 0.12, *β* = 0.13, *t* = 2.15, *p* < 0.05). Taken together, the six variables entered in block 2 explained an additional 6% of the variance in the outcome. Finally, in block 3, the publication pressure composite score was added and emerged as the strongest individual predictor of misconduct (*b* = 0.20, *β* = 0.34, *t* = 7.82, *p* < 0.01), controlling for all other explanatory variables in the model. As a group, all of the variables explained 21% of the variance in the misconduct score. Moreover, above and beyond the other explanatory variables already entered into the regression model, publication pressure explained an additional 10% of the variance in the misconduct score.

## Discussion

The present study examined the associations between HPE researcher characteristics, publication pressure, and the frequency of self-reported scientific misconduct and QRPs. Our findings suggest that the variables of age, number of publications, geographical location, work role, and publication pressure explain considerable variance in the frequency of researchers’ self-reported irresponsible research behaviours. Taken together, these findings provide the field with an evidence base from which to begin tailoring strategies to address scientific misconduct and QRPs in HPE.

Researchers have long described the pressure to publish as a contributor to the ‘dark side of science’ [18]. In biomedicine, researchers experiencing publication pressure are more likely to admit engaging in misconduct and QRPs [4, 8, 19, 20]. The findings reported here align with and extend this previous work. We found that both perceived publication pressure and the number of publications were associated with misconduct and questionable research conduct. To mitigate the pressure to publish, researchers have suggested modifying promotion and tenure structures, promoting and rewarding research transparency, and training senior researchers to act as responsible role models by demonstrating positive (and ethical) research practices [8, 9, 21]. Integrating such approaches in HPE may be beneficial; however, in many cases, HPE researchers are not directly assigned to a department of HPE, but rather are often members of clinical departments (e. g., Departments of Medicine, etc.). Therefore, for role modelling to have a positive impact on HPE researchers, such practices would likely need to be integrated at the institutional level.

To our knowledge, this is the first study to find a relationship between publication pressure and irresponsible research practices in HPE. Not only has publication pressure been connected with greater misconduct and QRPs, it has also been tied to researchers’ reluctance to share their work openly and to partner with other researchers and has been linked to higher levels of researcher burnout [22, 23]. Overall, these findings suggest that the topic of publication pressure may be ripe for further exploration in HPE.

Our findings indicate that older HPE researchers report misconduct and QRPs less frequently than young researchers. These results are compelling, especially considering the fact that older researchers have been involved in HPE research for much longer (*r* = 0.81) and thus have had more opportunities to act unethically. And yet, these results suggest they do not. Taken together, these findings provide some evidence for the idea that junior scientists may be particularly vulnerable to the lure of misconduct and QRPs in HPE or, alternatively, that junior researchers may simply be unfamiliar with responsible research practices [8]. In the biomedical and social sciences, this finding has led funders, such as the National Institutes of Health, to mandate that all grantees, including junior and senior researchers, complete responsible conduct of research training [24–26]. While such an approach is a positive step, it is worth nothing that most HPE research is unfunded, making it possible that our junior researchers may not be offered or exposed to such training. Furthermore, researchers have wrestled with the scope of this training, raising concerns that it often falls short of addressing the increasingly diverse nature of research [24, 27, 28]. As noted by Keune et al., when studying common HPE topics, such as examining residents and trainees, this training’s lack of diversity can mean many researchers are operating without guidance on how to navigate common ethical challenges within HPE [10].

Our results suggest that HPE researchers in Asia reported higher frequencies of irresponsible behaviours than researchers in North America. It is important to note, however, that this does not mean Asian researchers are more unethical than those from other regions, since the observed differences could have resulted from any number of factors, including differences in survey item interpretation, researchers’ awareness of their own behaviours, and respondents’ truthfulness. Nonetheless, in the wake of concerns that research misconduct in Asia is on the rise, multiple research teams have undertaken Asia-specific programs of research [29] to further examine the problem. For example, a team of Chinese scientists recently conducted a survey and found that 40% of research from China may be tainted by researcher misconduct [30]. In that particular study, the respondents attributed misconduct to lack of training and institutional oversight, and to extremely high pressures to publish. In studying geographical influence more broadly, Fanelli suggests that geographical variation in research misconduct may be related to the presence or absence of policies and procedures [8].

Respondents identifying primarily as researchers reported higher frequencies of QRPs and misconduct than those identifying primarily as clinicians or ’other’ (e. g., teachers or administrators). Although speculative, this finding could be related to the idea that the stakes tend to be much higher for investigators whose research outputs and published papers represent the primary measures of success. On the other hand, a clinician who conducts education research ‘on the side’, may be less incentivized to cut corners. Regardless of the explanation, we would advocate for future research to more closely explore this relationship. Related areas of exploration could include the impact of widespread and long-standing ethics training in clinical education [31, 32] in contrast to approaches taken (or not taken) by graduate programs in HPE. Future studies might also consider differences in the incentive structures and other systematic pressures for researchers as compared with clinicians.

### Limitations

Our study has several limitations. The curated sample had a modest response rate of 28.2% and therefore nonresponse bias is a real possibility. Although the wave analysis indicated that nonresponse bias likely had limited effects on our results, it is possible that certain groups, (e. g., senior researchers) are over-represented in our sample, thereby biasing the results. As such, we cannot say whether or not the findings presented here generalize to the entire population of HPE researchers around the globe. From the perspective of data structure, we had respondents nested within countries (and potentially nested within institutions). Thus, a multilevel modelling approach (e. g., hierarchical linear modelling) could have assisted in teasing out the sources of the variance in the outcome variable, as explained by the explanatory variables. However, we did not have a sufficient sample size at higher levels of unit of analysis, and we did not have institutional data; therefore, we could not conduct a multilevel analysis.

Responsible research behaviour is complex and context specific. We recognize that measuring such complex phenomena with self-report survey items has limitations. Moreover, self-report surveys can be sensitive to several types of response bias, including limitations of autobiographical memory and differences in interpretations of the meaning of questionable research behaviours. Notwithstanding these limitations, our findings align with the broader literature on ethical research practices. For example, Fanelli conducted a meta-analysis of 21 survey studies and concluded that 2% of respondents admitted to fabrication, a finding that exactly matches our results for this particular type of misconduct [33].

Lastly, we recognize that an individual’s decision to engage in research misconduct and/or QRPs is complex. Therefore, while we have identified a set of variables that seem to explain a fairly large proportion of variance in these behaviours, a much larger proportion of that variance (almost 80%) is still unexplained. What is more, the correlational nature of this study precludes us from making strong statements regarding causality and the specific reasons for the observed relationships; it also prevents us from making definitive suggestions for how we might mitigate such practices. As such, interventions intended to improve researcher ethics and conduct must be rigorously designed and tested in HPE settings.

## Conclusions

The results of this study suggest that a researcher’s age, number of publications, geographical location, and work role are associated with the frequency of self-reported misconduct and QRPs in HPE. We also found a positive association between perceived publication pressure and irresponsible research behaviours. Both scientific misconduct and QRPs have the potential to seriously damage the quality of our scientific work, as well as the public’s belief in its credibility. Ultimately, we hope these results can help academic leaders, journal editors, and others involved in educational research develop targeted measures to minimize scientific misconduct and QRPs in HPE.

## Caption Electronic Supplementary Material

Supplemental Histograms and Probability Plots

Questionable Research Practices Survey

List of all the journals included in the study
