# Supplementary material for: Factors associated with scientific misconduct and questionable research practices in health professions education
Source: Perspect Med Educ. 2019 Mar 26;8(2):74–82. doi: 10.1007/s40037-019-0501-x (PMC6468038; doi:10.1007/s40037-019-0501-x)
Supplement: Supplementary file 1 — Supplemental Histograms and Probability Plots [file 40037_2019_501_MOESM1_ESM.docx]

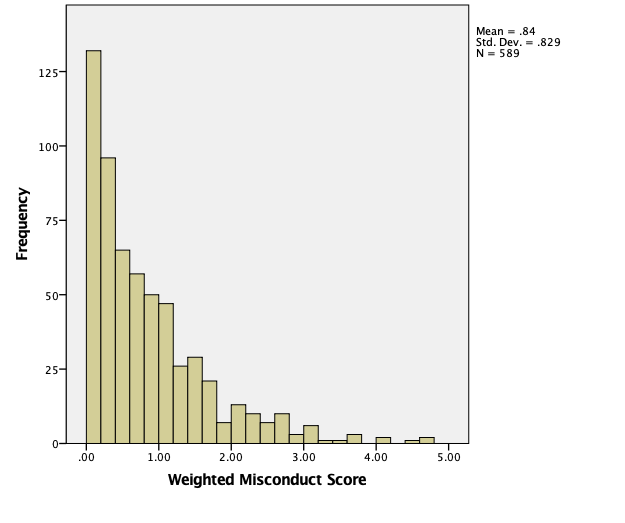


Supplemental Appendix 1

Figure 1. Histogram of the outcome variable “weighted misconduct score” before square root transformation


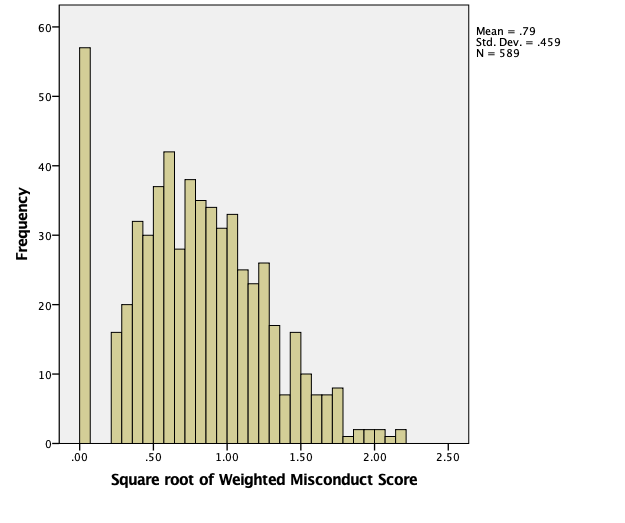


Figure 2. Histogram of the outcome variable “weighted misconduct score” after square root transformation


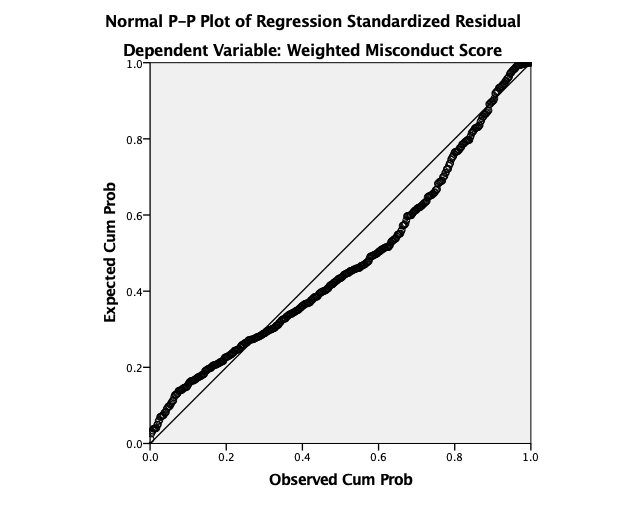


Figure 3. Normal probability plot of regression standardized residuals before transformation of the outcome variable “weighted misconduct score”


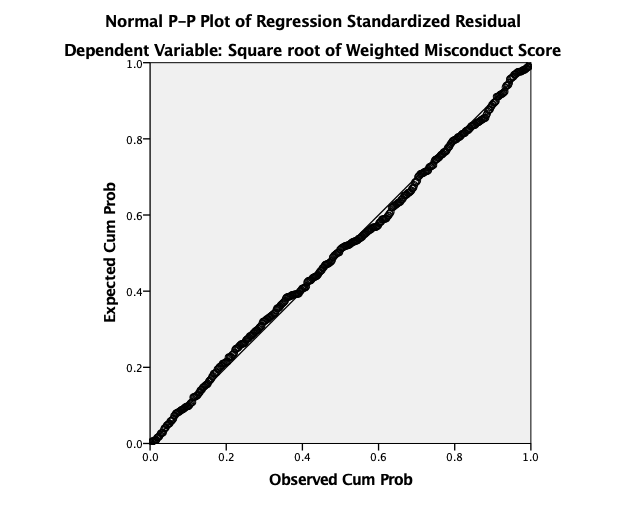


Figure 4. Normal probability plot of regression standardized residuals after transformation of the outcome variable “weighted misconduct score” (the same regression model applied as in Figure 3)
