# Supplementary material for: Factors associated with scientific misconduct and questionable research practices in health professions education
Source: Perspect Med Educ. 2019 Mar 26;8(2):74–82. doi: 10.1007/s40037-019-0501-x (PMC6468038; doi:10.1007/s40037-019-0501-x)
Supplement: Supplementary file 2 — Questionable Research Practices Survey [file 40037_2019_501_MOESM2_ESM.docx]

Supplemental Box 1: **List of all the journals included in the study**

| *Academic Medicine* |
| --- |
| *Advances in Health Sciences Education* |
| *African Journal of Health Professions Education* |
| *Annals of Academic Medicine of Singapore* |
| *BMC Medical Education* |
| *Clinical Teacher* |
| *Education for Health* |
| *Edumecentro* |
| *International Journal of Medical Education* |
| *Investigacion en Educacion Medica* |
| *Journal of Continuing Education in the Health Professions* |
| *Journal of Educational Evaluation for Health Professions* |
| *Journal of Graduate Medical Education* |
| *Korean Journal of Medical Education* |
| *Medical Education* |
| *Medical Education Online* |
| *Medical Teacher* |
| *Revista Brasileira de Education Medica* |
| *Revista de la Fundacion Educacion Medica* |
| *Teaching and Learning in Medicine* |

*Journals listed alphabetically.

Supplemental Digital Table

**Breakdown of response rates for health professions education researchers from across the World Health Organization’s six world regions (data based on curated sample)**

| Region | No. of respondents | No. of respondents + non-respondents | *Approximate Response rate (%) |
| --- | --- | --- | --- |
| North America | 172 | 827 | 20.8% |
| Europe | 115 | 496 | 23.2% |
| Africa and Middle East | 36 | 158 | 22.8% |
| Australia / New Zealand | 40 | 146 | 27.4% |
| Asia | 30 | 140 | 21.4% |
| South/Latin America and Caribbean | 17 | 73 | 23.3% |
| Total | 410 | 1840 | 22.3% |

*Note: the numbers and approximate response rates listed here are different from those reported in the text of the manuscript and Table 1 because these numbers (a) do not include emails that were returned as undeliverable, (b) do not include respondents who either reported their region as “other” or did not report a region, and (c) do not include respondents from the social media sample.
