# Supplementary material for: Factors associated with scientific misconduct and questionable research practices in health professions education
Source: Perspect Med Educ. 2019 Mar 26;8(2):74–82. doi: 10.1007/s40037-019-0501-x (PMC6468038; doi:10.1007/s40037-019-0501-x)
Supplement: Supplementary file 3 — List of all the journals included in the study [file 40037_2019_501_MOESM3_ESM.docx]

Questionable Research Practices Survey

Thank you for your interest in our survey!

We, Erik Driessen, Tony Artino, and Lauren Maggio, are inviting authors, such as yourself, who published HPE research in 2016 to participate in this health professions education (HPE) study. Please read the below information sheet carefully and feel free to contact us if you have additional questions by using the email addresses below.
 **Study purpose:**

This study seeks to determine the prevalence of questionable research practices (QRPs) in HPE to inform educators, practitioners, and journal editors. In doing so, we hope the HPE community might be better positioned to take evidence-informed action, should the results indicate a need for such action. This research project includes this administration of an online survey and the aggregate analysis of collected survey data. We aim to publish our findings in a peer-reviewed journal. 
 **Expectations:**If you agree to participate, we will ask you to complete an online survey. The survey can be completed on a computer or mobile device. We estimate the survey will take approximately 12 minutes. The survey, includes 43 Likert-type questions that ask you to indicate the prevalence of QRPs in your research. The survey also includes 24 additional items about publication pressure and basic demographic information (e.g., professional degrees earned, gender). No preparation is necessary for the survey.
 **Risk:**
There is no direct advantage for you in participating in this study. However, we believe it might provide a better understanding of QRPs and thus may have advantages for the field of HPE research. It is up to you to decide whether or not to participate in the study. Participation is voluntary. If you do participate in the study, you can always change your mind and stop participating at any time during the study, without giving a reason. If you decide to stop the study, any data collected will be deleted and not used for this study. Whether you participate or not, there are no negative consequences for you. You will not be paid for your participation in this study. A potential disadvantage of participation is the time (approximately 15 minutes) it will take to complete the survey. 
 **Data storage:**Data collection will begin upon clicking the below link to the survey. Each time you reach the end of a survey page and elect to continue, responses from that survey page will be submitted and stored. No personal identifiable information will be collected (i.e., we will not collect your name, email, or IP address). Data will be stored anonymously in Maastricht University’s Qualtrics account and is not traceable to you. Also, your responses will be analyzed in combination with those of other respondents. Only Drs. Driessen, Artino and Maggio will have access to the anonymous data. All data will be reported in aggregate in any reports or publications.

If you participate in this study, you consent to the data being stored for 10 years after ending the study for further analysis within context of this project. You cannot participate in this study if you do not give permission for this data storage (by consenting below). After 10 years, the data will be destroyed. 

This study has been approved by the Ethical Review Board Committee of the Netherlands Association for Medical Education (Dossier #937)

Please don't hesitate to contact us directly if you have any questions about the research or this survey or would like further information. We can be contacted at the following email addresses and phone numbers: Erik (e.driessen@maastrichtuniversity.nl; phone: 31(0)43-3885774)
  
We appreciate your time and expertise! 
Erik, Tony and Lauren

If you consent to taking this study, please read and check yes below:
  
I have read the above information sheet for participants. I have had the opportunity to contact the investigators ask additional questions. My questions have been sufficiently answered. I have had enough time to decide whether to participate or not.
  
I know that participation is entirely voluntary. I am aware of my right to withdraw or end my participation from the study at any time. I do not need to justify that decision.
  
I know that certain people have access to my data. These people are listed in this information sheet. I am entitled to inquire and look into how my data are stored.
  
I consent to my data being used in the way and for the purpose stated in the information sheet. If for any reason my data would be used for research with another objective, I will be informed and again be asked to consent.
  
I consent to my data being stored for another 10 years after ending this study to permit further analysis within the context of this study.

I consent to participate in this study.

- Yes
- No

**Research Practices:** In your work as an HPE researcher, **how often have you engaged in any of the following behaviors**, even if it has been only on a single occasion? If applicable, please consider your experiences with both quantitative and qualitative research.

|  | Never | Once | Occasionally | Sometimes | Frequently | Almost always | Not applicable to my work |
| --- | --- | --- | --- | --- | --- | --- | --- |
| Conducted a human-subjects research study without ethics approval (i.e., without institutional review board [IRB] approval) |  |  |  |  |  |  |  |
| Circumvented one or more aspects of human-subjects ethics rules (i.e., IRB rules) |  |  |  |  |  |  |  |
| Collected course or curriculum data under the guise of “program evaluation” without human-subjects ethics (IRB) approval with the ultimate intent of using the data for research purposes |  |  |  |  |  |  |  |
| Inappropriately stored sensitive research data (e.g., data that contains personally identifiable information) |  |  |  |  |  |  |  |
| Inappropriately emailed sensitive research data (e.g., data that contains personally identifiable information) |  |  |  |  |  |  |  |
| Stopped collecting data earlier than planned because the results already reached statistical significance, without formal stopping rules |  |  |  |  |  |  |  |
| Fabricated data |  |  |  |  |  |  |  |
| Pressured a student or other subordinate to be a study participant in your research |  |  |  |  |  |  |  |
| Used students or residents as research subjects without informing the overseeing dean, program director, or other pertinent official |  |  |  |  |  |  |  |

|  | Never | Once | Occasionally | Sometimes | Frequently | Almost always | Not applicable to my work |
| --- | --- | --- | --- | --- | --- | --- | --- |
| Deleted data before performing data analysis without disclosure |  |  |  |  |  |  |  |
| Ignored a colleague’s use of flawed data |  |  |  |  |  |  |  |
| Ignored a colleague’s questionable interpretation of data |  |  |  |  |  |  |  |
| Reported a downwardly rounded p-value (e.g., reporting that a p-value of .054 is less than .05) |  |  |  |  |  |  |  |
| Misrepresented a participant’s words or writings |  |  |  |  |  |  |  |
| Decided whether to exclude non-outlier data after looking at the impact of doing so on the results |  |  |  |  |  |  |  |
| In a qualitative study, failed to report disconfirming examples or cases that weaken your conclusions |  |  |  |  |  |  |  |
| Collected more data after seeing that the results were almost statistically significant |  |  |  |  |  |  |  |
| To confirm a hypothesis, selectively deleted or changed data after performing data analysis |  |  |  |  |  |  |  |
| Reported an unexpected finding as having been hypothesized from the start |  |  |  |  |  |  |  |
| Concealed results that contradicted your previous findings or convictions |  |  |  |  |  |  |  |

|  | Never | Once | Occasionally | Sometimes | Frequently | Almost always | Not applicable to my work |
| --- | --- | --- | --- | --- | --- | --- | --- |
| Claimed you used a particular qualitative research approach appropriately (e.g., grounded theory) when you knowingly did not |  |  |  |  |  |  |  |
| Claimed you used a particular qualitative research technique appropriately (e.g., saturation, triangulation) when you knowingly did not |  |  |  |  |  |  |  |
| Spread study results over more papers than is appropriate (so-called “salami slicing”) |  |  |  |  |  |  |  |
| Deliberately failed to mention important limitations of a study in the published paper |  |  |  |  |  |  |  |
| Deliberately failed to mention an organization that funded your research in the published paper |  |  |  |  |  |  |  |
| Inappropriately modified the results of a study due to pressure from a research advisor or other collaborator |  |  |  |  |  |  |  |
| Inappropriately modified the results of a study due to pressure from a funding agency |  |  |  |  |  |  |  |
| Failed to disclose relevant financial or intellectual conflicts of interest |  |  |  |  |  |  |  |
| Used someone else’s ideas without their permission or proper citation |  |  |  |  |  |  |  |
| Used sections of text from another author’s copyrighted material without permission or proper citation |  |  |  |  |  |  |  |
| Used sections of text from your own publications without proper citation (so-called “self-plagiarism”) |  |  |  |  |  |  |  |

|  | Never | Once | Occasionally | | Sometimes | | Frequently | | Almost always | | Not applicable to my work | |
| --- | --- | --- | --- | --- | --- | --- | --- | --- | --- | --- | --- | --- |
| Selectively cited certain papers just to please editors or reviewers |  |  |  |  | |  | |  | |  | |  |
| Cited articles and or materials that you have not read |  |  |  |  | |  | |  | |  | |  |
| Selectively cited your own work just to improve your citation metrics |  |  |  |  | |  | |  | |  | |  |
| Reused previously published data without disclosure (co-called “duplicate publication”) |  |  |  |  | |  | |  | |  | |  |
| Used confidential information obtained as a reviewer or editor for your own research or publications |  |  |  |  | |  | |  | |  | |  |
| Refused to share data with legitimate colleagues |  |  |  |  | |  | |  | |  | |  |
| Added one or more authors to a paper who did not qualify for authorship (so-called “honorary authorship”) |  |  |  |  | |  | |  | |  | |  |
| Accepted authorship for which you did not qualify (so-called “honorary authorship”) |  |  |  |  | |  | |  | |  | |  |
| Demanded authorship for which you did not qualify (so-called “honorary authorship”) |  |  |  |  | |  | |  | |  | |  |
| Omitted a contributor who deserved authorship |  |  |  |  | |  | |  | |  | |  |
| Submitted (or re-submitted) a manuscript or grant application without consent from one or more of the author |  |  |  |  | |  | |  | |  | |  |
| Submitted the same manuscript to multiple journals at once (so-called “duplicate” or “double submission”) |  |  |  |  | |  | |  | |  | |  |

**Publication Pressure:** These items address publication pressure. Please indicate the extent to which you agree or disagree with the following statements, as they relate to your particular HPE context.

|  | Completely disagree | Somewhat disagree | Neither agree nor disagree | Somewhat  agree | Completely agree |
| --- | --- | --- | --- | --- | --- |
| Without publication pressure, my scientific output would be of higher quality |  |  |  |  |  |
| My colleagues’ assessments of me, based on my publications, are stressful |  |  |  |  |  |
| Publication pressure strains my relationships with fellow researchers |  |  |  |  |  |
| I suspect that publication pressure leads some colleagues (whether intentionally or not) to inappropriately manipulate their data |  |  |  |  |  |
| Publication pressure leads me to have serious doubts about the validity of HPE research results |  |  |  |  |  |
| In my opinion, the pressure to publish scientific articles has become too high |  |  |  |  |  |
| My colleagues judge me mainly on the basis of my publications |  |  |  |  |  |
| I cannot share innovative research proposals with my colleagues for fear of those ideas being stolen |  |  |  |  |  |
| Publication pressure harms science |  |  |  |  |  |

**Demographics:**
What is your gender?

- Male
- Female

What is your age? [Drop-down menu]

- 1 to 100

In which country or region do you primarily work? [Drop-down menu]

▼ Africa ... Other

- Africa
- Asia
- Australia/New Zealand
- Canada
- Caribbean
- Europe (not including the UK
- Middle East
- South/Latin America
- United Kingdom
- United States
- Other

What is your current academic rank or position title? (Select one)

▼ Medical Student (1) ... Not Applicable (14)

- Medical Student
- Resident
- Graduate/PhD Student
- Fellow
- Postdoc
- Instructor
- Lecturer
- Assistant Professor
- Associate Professor
- Professor
- Professor Emeritus
- Staff
- Other
- Not Applicable

Which degree(s) do you hold? (Check all that apply)

- Bachelor’s degree (BS, BA, BSN, etc.)
- Master’s degree (MA, MS, MSW, MPH, MSN, etc.)
- Professional medical degree (MD, DO)
- Doctoral degree (PhD, EdD, DrPH, etc.)
- Other professional degrees (JD, PA, DVM, etc.)

For the highest degree you selected above, what is your primary area of study? (Select one)

- Basic Science
- Clinical Science
- Social Science/Education
- Humanities
- Other: ________________________________________________

What is your primary work role? (Select one)

- Clinician
- Administrator or Program Director
- Teacher (clinical or classroom setting)
- Researcher
- Other: ________________________________________________

Which of the following best describes the context in which you work? (Check all that apply)

- Undergraduate Medical Education (UME)
- Graduate Medical Education (GME)
- Continuing Medical Education (CME)

In a typical work week, approximately what percentage of your work time do you spend on health professions or medical education research activities, including writing up your research (please report your answer as a percentage)?

________________________________________________________________

How many years have you been involved in health professions or medical education (in any capacity)?

________________________________________________________________

How many years have you been involved in conducting research in health professions or medical education?

________________________________________________________________

In thinking about your primary research activities, how would you characterize your work (Select one)?

- I am a quantitative researcher
- I am a qualitative researcher
- I am a mixed-methods researcher

In total, approximately how many peer-reviewed publications have you authored or co-authored?

________________________________________________________________

If you have any other comments related to questionable research practices in HPE, or comments about this questionnaire, please share those here:

________________________________________________________________

________________________________________________________________

________________________________________________________________

________________________________________________________________

________________________________________________________________

Note that by clicking the below "next" button you will submit your questionnaire responses and be unable to return to the questionnaire.

Next

We thank you for taking time to complete this survey. 
Your responses have been recorded.

[Powered by Qualtrics](http://www.qualtrics.com/)
